# Immediate Effects of Focal Muscle Vibration on Squat Power and Velocity in Amateur Athletes: A Randomized Controlled Trial

**DOI:** 10.3390/jfmk10010060

**Published:** 2025-02-10

**Authors:** Sergi Rodríguez-Rodríguez, Max Canet-Vintró, Carlos López-de-Celis, Zhifan Shen-Chen, Iván Caballero-Martínez, Erik García-Ribell, Jacobo Rodríguez-Sanz

**Affiliations:** 1 Department of Medicine, Faculty of Medicine and Health Sciences, Universitat Internacional de Catalunya, 08195 Barcelona, Spain; srodriguezr@uic.es (S.R.-R.); maxcanet44@uic.es (M.C.-V.); erik.garcia@uic.es (E.G.-R.); jrodriguezs@uic.es (J.R.-S.); 2 Actium Functional Anatomy Research Group, 08195 Sant Cugat del Vallés, Spain; zhifanshen@uic.es (Z.S.-C.); ivancaballero@uic.es (I.C.-M.); 3 Fundació Institut Universitari per a la Recerca a l’Atenció Primària de Salut Jordi Gol i Gurina (IDIAPJGol), 08007 Barcelona, Spain; 4 Department of Physiotherapy, Faculty of Medicine and Health Sciences, Universitat Internacional de Catalunya, 08195 Barcelona, Spain

**Keywords:** focal vibration, squat, power, velocity, performance, athletes

## Abstract

**Background:** Squat exercises are widely recognized for their ability to improve sports performance. Recent advancements in force/velocity profiling have highlighted the importance of power and velocity in explosive movements. While various training methods have been applied to enhance these parameters, their effectiveness remains inconsistent. Focal vibration has emerged as a potential intervention, yet its impact on squat performance has not been extensively explored. The aim was to evaluate the effectiveness of focal vibration with voluntary contractions on power/velocity during a squat exercise in amateur athletes in comparison with voluntary contraction in isolation. **Methods:** A double-blind, randomized clinical trial with 72 amateur athletes. Velocity, power, muscle activity, perceived effort, and clinical change were measured. The experimental group received focal muscle vibration therapy (100–180 Hz) combined with voluntary contraction, while the sham group underwent identical procedures and focal muscle vibration without the vibrating head, close to the muscle belly but without touching the skin. **Results:** Statistically significant improvements in the experimental group were found for mean power (*p* < 0.001; ES = 0.08), peak velocity (*p* < 0.010; ES = 0.42), and mean velocity (*p* < 0.001; ES = 0.66) during the squat. Between-group analysis favored the experimental group in peak power (*p* < 0.049; ES = 0.65), mean power (*p* < 0.034; ES = 1.42), peak velocity (*p* < 0.024; ES = 0.095), and mean velocity (*p* < 0.002; ES = 1.67). **Conclusions:** Focal muscle vibration (100–180 Hz) combined with active muscle contraction significantly enhances power and velocity during squat exercises in amateur athletes.

## 1. Introduction

Sports performance is a complex mix of biomechanical functions which depends on a complex interaction of variables [1]. Every athlete wants to perform well, to win, and to improve their performance [2]. Among the most used exercises to train and improve performance, squat exercise stands out in the literature [3]. This study [3] has described that it is an effective exercise for strengthening the lower limbs, protecting against injuries and improving sports performance. Moreover, this type of explosive exercise involves the largest and strongest muscles of the body (quadriceps, hamstrings, gluteus maximus, triceps surae, and erector spinae) and demands a coordinated multi-joint (spine, hip, knee, and ankle) movement [3,4].

In these explosive exercises, power and velocity relationships are the ideal parameters to study to determine the conditions of muscular work required [5,6,7]. Recent advancements in power profiling have allowed coaches to better assess athletes’ neuromuscular performance [8], as it is an important indicator of successful performance in a variety of sports [9]. On the other hand, velocity enables the efficient execution of those movements. Zemková and associates [10] showed that athletes who generate higher power and have also more velocity tend to outperform peers in tasks like sprinting and jumping. These attributes are tied to neuromuscular adaptations and muscle fiber composition, particularly in sports demanding high-force production in a short timeframe [10].

Nowadays, to improve performance, several methods such as short-term periodization training [11], eccentric-overload training [12], blood flow restriction training [13], resisted sprint training [14], or whole-body vibration training have been applied [15], but the efficacy in producing significant improvement in performance-related muscle parameters in elite athletes is still in doubt, showing results without significant differences in strength, power, and flexibility [16].

In this sense, new modalities have been investigated in recent years to reduce monotony, increase treatment efficacy, and improve performance [17]. Focal muscle vibration using high frequencies (>100 Hz) has been shown to have an immediate effect on increasing muscle activity and metabolic response [18]. This type of vibration allows for allocating a more localized stimulus to a single muscle or specific muscular group, with more possibilities to be applied during an exercise [16]. The neurophysiological mechanism through which vibratory stimulation operates has been attributed to the tonic vibratory reflex [19]. This mechanism is stimulated by a sequence of rapid muscle stretching that occurs when applying vibration, triggering muscle spindles and thereby causing an involuntary production of strength [19,20].

Even though, when vibration is used in isolation in different leg muscles and at a frequency < 100 Hz, Rogan and associates [21] did not report in their systematic review any changes in dynamic muscle strength and power. Furthermore, compared to groups performing conventional types of exercise, vibration therapy had only small and non-significant changes [21]. For this reason, the local vibration intervention applied during a voluntary contraction has been recommended for improving performance, suggesting that it promotes increased motor unit activation and firing frequency, increased excitation of the alpha motor neurons through the muscle spindle system, and changes in corticospinal excitability [22]. To date, the effect of focal vibration application on squat performance has not been studied yet. For this reason, it was hypothesized that adding focal vibration during voluntary contractions would have positive effects on power/velocity during a squat exercise, consequently improving squat performance.

This study aimed to evaluate the effectiveness of focal vibration with voluntary contractions on power/velocity during a squat exercise in amateur athletes in comparison with voluntary contraction in isolation.

## 2. Materials and Methods

### 2.1. Study Design

This study was a double-blind, randomized clinical trial carried out between November and December 2024. The participants were divided into two groups; both did a muscular activation protocol involving lower-limb muscular voluntary contractions. Furthermore, at the same time, the experimental group (EG) received a focal vibration treatment (100–180 Hz), and the sham group (SG) received a placebo treatment [23]. Random assignment was performed with a computerized list randomizer (https://www.random.org/lists/ (accessed on 1 November 2024)), which generates a random list for the experimental group and sham group.

The study protocol was registered under ClinicalTrials.gov identifier NCT06671951. The study protocol was approved by the local ethics committee (CBAS-2023-05) and complies with the principles of the Declaration of Helsinki [24], and the Consolidated Standards of Reporting Trial (CONSORT) guidelines were followed throughout the study.

### 2.2. Simple Size Calculation

The sample size was calculated based on the study by Gallego-Sendarrubias and associates [25] for the variable mean velocity during the squat. The GRANMO V.7.12 statistical program was used with a common standard deviation of 0.3, a minimum difference to be detected of 0.2, an α risk of 0.05, and a β risk of 0.2. A total of 72 subjects (36 for each group) were calculated.

### 2.3. Participants

A total sample of 72 (46 men; 26 women) amateurs’ athletes were recruited from Universitat Internacional de Catalunya for this study, with a mean age of 24.69 years ± 5.2 (Men 25.19 ± 5.5; Women 23.76 ± 4.7).

Inclusion criteria were as follows: (a) healthy athletes training 3 days a week and at least one of them had to involve lower-limb strength; (b) to be familiar with squat exercise and the technique of it; and (c) to have signed the informed consent. Exclusion criteria included the following: (a) participants who reported taking anabolic drugs or any other medical drug treatment that could interfere with the measurements were excluded from the study; (b) having ever received focal vibration treatment; (c) suffer from chronic diseases or ongoing injuries; and (d) having any type of lower limb or back injury.

### 2.4. Measurements

The primary outcome measures in this study were the mean velocity and mean power during the squat set. Secondary outcomes measures were maximal velocity and maximal power; furthermore, we measured muscle activity, the scale of perceived effort, and the global rating of change scale (GROC-Scale).

### 2.5. Variables

#### 2.5.1. Velocity and Power During Squat

A linear position transducer (Vitruve System SL, Málaga, Spain) was used to measure the power and velocity during the squat. The tether device was attached to the right side of the barbell around the widest part of the collar on the inside and this converts the subject’s movement with the barbell into an electrical signal that will record the velocity and power of the exercise. Subjects performed 7 repetitions at 0.85 m/s at the maximum possible velocity with the optimal load to generate the maximum power [26,27]. Both the mean and maximal velocity were calculated, and the same results were obtained with power. In this context, linear transducers have been shown to offer greater accuracy (5%) and reliability (ICC: 0.752; 95% CI: 0.548–0.855) compared to other devices for velocity-based training (VBT) measurement [27,28], ensuring the precision of the data collected during the experiment.

#### 2.5.2. Scale of Perceived Effort During Squat

Just after the end of the series of 7 repetitions, the sensation of effort perceived by the athlete was measured with the modified BORG scale (0–10), with 0 being the lowest perceived effort and 10 being the highest effort [29].

#### 2.5.3. Participant’s Rating of Clinical Change

At the end of the free bar squat after the warm-up and the intervention, the subject was asked if he/she felt improvement or worsening between the first time and the last time he/she performed the test. The participant’s rating of clinical change was evaluated with the GROC scale [30]. This is a scale of 15 items, of which 7 are improvement and 7 are deterioration, and with 1 central item with no clinical change. Values from the fourth item of improvement or deterioration were considered clinically significant, values between the three items of improvement and the three items of deterioration were considered as no clinically significant changes [31]. The test–retest reliability has proven to be good (ICC 0.90) [32].

#### 2.5.4. Muscle Activity

Surface electromyography (sEMG) was used to evaluate the muscle activity of the back and lower limbs during the squatting task. In a related study examining a similar movement such as vertical jump, among other variables, Fauth and associates [33] discovered all ICC values to be above 0.80, with the majority exceeding 0.90. Furthermore, the validity of the sEMG mDurance^®^ system (mDurance Solutions SL, Granada, Spain) was confirmed for recording muscle activity during a functional task (ICC = 0.916; 95% CI = 0.831–0.958) [34]. The muscles assessed were vastus medialis (VM), vastus lateralis (VL), rectus femoris (RF), biceps femoris (BF), gluteus maximus (GM), and erector spinae longissimus (ESL). Data were obtained for the dominant limb defined as the one preferred to kick a ball [35].

The mDurance^®^ system consists of three parts: (a) A Shimmer3 sEMG unit (Realtime Technologies Ltd., Dublin, Ireland). This unit is a bipolar surface electromyography sensor for acquiring muscle activity. Each Shimmer3 has two channels, with a sampling rate of 1024 Hz. Shimmer3 applies a bandwidth of 8.4 Hz, and the sEMG signal resolution is 24 bits and has an overall amplification of 100 to 10,000 *v*/*v* [36]; (b) the mDurance Android application, which receives the data from the Shimmer3 and sends it to a cloud service [37]; and (c) the mDurance cloud service where the data is stored, filtered, and analyzed [37]. For the processing and filtering of raw data, both isometric and dynamic tests were filtered using a fourth-order Butterworth bandpass filter with a 20–450 Hz cut-off frequency. The signal was smoothed using a window size of 0.025 s root mean square (RMS) and an overlapping of 0.0125 s between windows [37]. The RMS was the principal variable recorded for muscle activity, measured with microvolts μV.

The participants’ skin was cleaned with alcohol and dried before the electrodes were placed. If hair impeded the correct adhesion of the electrodes to the skin, the particular site was shaved. Self-adhesive 5 × 5 cm Valutrode^®^ (Axelgaard Manufacturing Co., Ltd., Fallbrook, CA, USA) surface electrodes were placed on the muscle bellies according to the SENIAM project recommendations [38] and with an interelectrode distance of 20 mm [37]. VM electrodes were placed at 80% on the line between the anterior superior iliac spine and the joint space in front of the anterior border of the medial collateral ligament of the knee, with an orientation almost perpendicular to this same line for the belly muscle. VL electrodes were placed between the line from the anterior superior iliac spine to the lateral side of the patella, and they were placed 2/3 s following the direction of the belly muscle. The electrodes for the RF were also placed on the midpoint between the anterior superior iliac spine and the patella midpoint for the belly muscle. Reference electrodes were placed at the patella midpoint and anterior superior iliac spine. Electrodes were placed in the middle of the line between the sacral vertebrae and the greater trochanter, in the same direction of the GM line, for the BF on the line between the ischial tuberosity and the lateral epicondyle of the tibia, they were also placed at the halfway point. Finally for the ESL, the participant laid prone, and the electrodes were respectively placed at a 2-finger width lateral from the processus spinalis of L1 and 1-finger width medial from the line from the posterior–superior iliac spine to the lowest point of the lower rib, at the level of L2.

For the muscle activity normalization and getting the 100% of maximal voluntary contraction on each muscle, we considered the mean RMS of the 7 repetitions performed in the baseline squat set.

### 2.6. Intervention

A physical therapist took the test–retest measurements, and the intervention was carried out by another physical therapist familiar with focal vibration therapy treatments. Both therapists have over a decade of experience in physical therapy. The intervention was administered individually in the facilities of the Universitat International de Catalunya. Both groups of participants were given a single 20 min session.

The athletes were asked what their 1RM (repetition maximum) was, to calculate the weight with which they would perform the repetitions at a velocity between 0.80–0.85 m/s, and this velocity corresponds to 60% of their 1RM [39]. This velocity is the one at which the athlete can generate greater power considering the strength–velocity profile [39,40]. Once the weight that the athlete had informed us was set, it was checked with the linear encoder. In case the athlete did not know 60% of their 1RM, they were given an appointment a week before to make the approximation.

Each participant performed a 5 min warm-up focused on lower-limb and back mobility [41]. The specific exercises were self-selected, allowing participants to choose movements they were most accustomed to and familiar with based on their regular training routines. Then, we established a protocol for approaching 2 sets of squats; the first approach set was 5 repetitions at 50% of 1RM and the second set was 3 repetitions at 75% of 1RM [21]. After the approach series, the pre-intervention measurement was carried out at 60% of 1RM, where the athletes performed 7 repetitions at the highest possible velocity [26,40].

The squats were performed with the bar centered between the shoulders, with the toes pointing slightly outwards and the feet shoulder-width apart. The participants were instructed to perform a controlled lowering movement on the knee, ankle, and hip joints with the back of the thighs parallel to the ground. The 90° knee angle was controlled with a bench to prevent further descent, taking into account the height and leg length on each patient. They were asked to ascend as fast as possible to an upright position [41].

After resting for 5 min, voluntary contractions with dynamic exercises accompanied by focal vibration began, which, depending on the previously assigned group, were in the form of a treatment or sham. The experimental group received a single treatment with the focal vibration machine (V-Plus Wintecare^®^, Wintecare SA, Chiasso, Switzerland), while the subject carried out active contractions. The positioning of the vibration heads was determined by the type of exercise, and those were fixed by a webbing designed by the manufacturer to keep the vibration head stable on the muscle. The vibration program was set to a predeterminate mode of 10 s of vibration and 3 s of rest. The frequency increases automatically in 10 Hz steps from 100 Hz to 180 Hz, and then progressively decreases again, in a cyclical way. The patient’s perception of pain was monitored throughout the treatment, stopping the treatment if the patient complained of pain.

The first application with focal vibration consisted of a bilateral lower-back extension, with 2 sets of 10 repetitions, and the subject rested for 15 s between sets. Two focal vibration channels were placed on the lower-back muscle bellies. The second application with focal vibration involved activating the hip extensor muscles by performing 2 sets of 8 repetitions with each leg of a unilateral gluteal bridge. The therapist applied two headers in the gluteus maximus and biceps femoris muscle bellies.

The third application consisted of 2 sets of 10 repetitions of the last 30° of the knee extension, at the beginning of the exercise, an isometric contraction for 5 s, and then completing the full knee extension. The subject rested for 15 s between sets, and when the first leg finished the 3 sets, it rested for 1 min (time in which the contralateral leg was doing the same exercise). Three focal vibration channels were placed on the muscle bellies of the rectus femoris, vastus medialis, and vastus lateralis.

For the last focal vibration application, focal vibration heads were placed on the gluteus maximus, and lateral and medial vastus. Participants performed 2 sets of 6 repetitions of the last third of the squat (30°) resisted at the start of the movement by the therapist.

**Figure 1:**
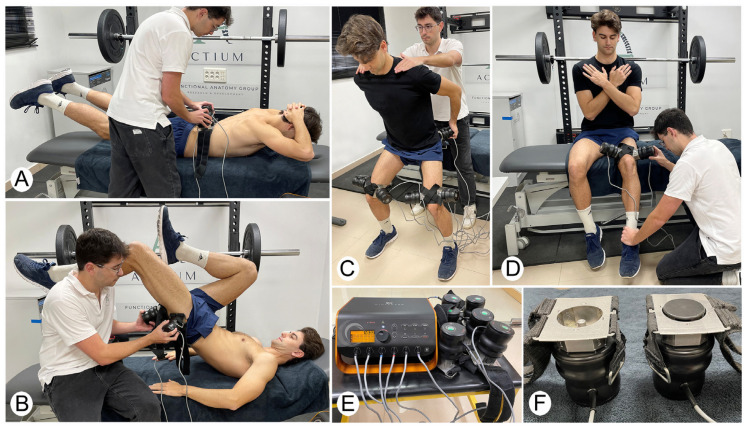
Intervention protocol for athletes at the intervention group or sham group: (**A**) bilateral lower-back extension, (**B**) hip extension, (**C**) knee extension, (**D**) 30° squat, (**E**) focal vibration machine, and (**F**) focal vibration heads (left for sham group; right for intervention group).

For the sham group, the same procedures were performed as in the experimental group, with the same active exercises, same physiotherapist procedures, and same placement of focal vibration channels. The model of Toscano et al. [40] was followed to apply the placebo. The focal vibration channels were placed without the vibrating head, close to the muscle belly but without touching the skin. The focal vibration machine was turned on, so in this condition, patients were only subject to the faint buzzing sound of the vibrator. See the intervention in Figure 1.

**Figure 2:**
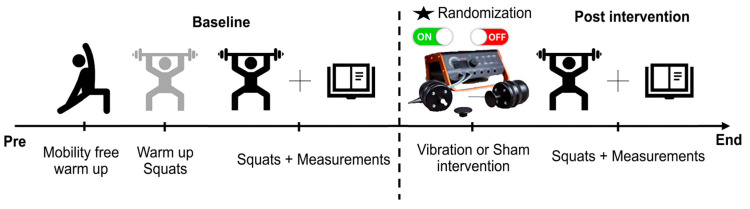
Schematic study timeline diagram.

After the intervention, the participants re-tested 7 reps with the same weight as the previous series. An experimental timeline diagram is shown in Figure 2, as a schematic representation of the whole study procedures.

### 2.7. Statistical Analysis

Statistical analysis was conducted with the SPSS 23.0 package (IBM, Armonk, NY, USA). There was no loss of follow-up in the study. The mean and standard deviation were calculated for each variable. The Kolmogorov–Smirnov test was used to determine a normal distribution of quantitative data (*p* > 0.05). Within- and between-group differences were analyzed using a linear mixed model. If the assumption of sphericity was violated, the Greenhouse–Geisser correction was utilized for interpretation. The effect size was calculated with Cohen’s d coefficient. Cohen’s coefficients were interpreted as follows: large effect sizes, d > 0.8; moderate effect sizes, d = 0.5–0.79; and small effect sizes, d = 0.2–0.49. The level of significance was set at *p* < 0.05.

## 3. Results

**Figure 3:**
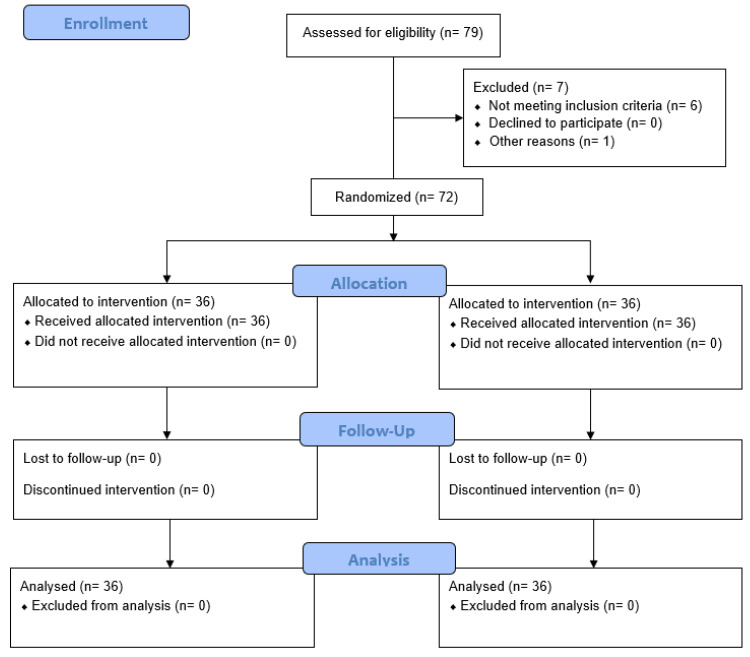
CONSORT (Consolidated Standards of Reporting Trial) flow diagram.

During November and December, 72 participants (EG, n = 36; SG, n = 36) were recruited. All the participants met all the eligibility criteria and agreed to participate. Then, the participants were randomly assigned to each group and received their assigned treatment. Enrolment and exclusions after randomization can be seen in Figure 3. The demographic characteristics of the sample are summarized in Table 1. No adverse events or side effects were reported for any participant.

Statistically significant differences were found in the group–time interaction analysis for all the variables. Statistically significant differences in the experimental group during the squat were found; this group improved the mean power (*p* < 0.001; η2 = 0.475), peak velocity (*p* < 0.010; η2 = 0.091), and mean velocity (*p* < 0.001; η2 = 0.504). In the sham group, a statistically significant difference was found only in the peak velocity (*p* < 0.016; η2 = 0.080); this group decreased their peak velocity during the second squat set. Table 2.

In the between-group analysis, statistically significant differences were found in favor of the experimental group in peak power (*p* < 0.049; η2 = 0.054), mean power (*p* < 0.034; η2 = 0.063), peak velocity (*p* < 0.024; η2 = 0.070), and mean velocity (*p* < 0.024; η2 = 0.130) (Table 3).

Regarding the Borg scale, no statistically significant differences were found. The sham group showed higher but minimal values (3.38 Pre; 3.96 Post) compared to the experimental group (3.22 Pre, 3.90 Post). There were no statistically significant changes between the Groc scales; however, 16.7% of the experimental group showed a majority versus 11.1% of the sham group. Only one subject in the sham group (2.8%) reported a worsening.

No statistically significant differences were found in the group–time interaction analysis for muscle activity in any muscles analyzed (Table 4).

## 4. Discussion

This study aimed to evaluate the effectiveness of focal muscle vibration with active muscle contraction on power/velocity during a squat exercise in amateur athletes. We have found statistically significant differences in post-treatment measurements between groups in all the variables, in favor of the experimental group compared to the sham group, as hypothesized in the introduction. The purpose of these results is to facilitate new strategies for therapists and trainers to improve the performance of their patients while performing squats. This goal translates into more efficient squat training in clinical practice.

Over the recent past, establishing additional training protocols over a traditional training routine has been a key focus of sports and conditioning experts [41]. The vibration training method has recently emerged as an effective and convenient technique in improving neuromuscular performance especially when combined with a strength training program [42]. A recent study by Azzollini and associates [43] has found an increase in muscle strength while introducing segment-body vibration to the tendons of the same muscles, producing an improvement in the synchronization of neuronal activity between the muscles and an increase in maximum force. In the present study, similar results were found for the experimental group that received focal vibration at a high frequency (100–180 Hz), with active muscle contraction in the parameters of mean power, peak power, and peak and mean velocity during the squat. These results also agree with relevant data from the literature [44], where using focal vibration with a frequency of 100–180 Hz promotes long-term potentiation and results in an immediate and sustained change in synaptic responsiveness and reorganization of the synaptic pathway, resulting in an increase in muscle strength [44].

An important factor to take into consideration when analyzing the data from this study is that it was conducted on amateur athletes. Some authors [44,45] have related that differences in training status could be an important moderator variable for acute performance enhancements (e.g., muscle power) following activities with higher effectiveness in amateur athletes than in trained ones. Added to this, there is compelling evidence that longer vibration exposure times of a 20–30 min duration can decrease subsequent EMG activity and muscular performance in upper and lower-limb muscles [46]. Unlike the study mentioned before [46], an important factor in our study is that the application of focal vibration plus active contraction was intermittent during the 20 min, with high-intensity strength exercises and very short periods accompanied by 100–180 Hz intermittent focal vibration that was applied only during active exercise. Enoka RM described in his book that intermittent and variable stimulus can induce greater muscle adaptations because of the differential activation of muscle fibers and prevention of muscle tissue habituation, maximizing neuromuscular activation and causing greater hypertrophy [47]. Moreover, several studies [20,48,49,50] have analyzed the effects of a specific intervention, called muscle vibration, with focal stimulation characterized by a frequency of 100 Hz, amplitude of 0.2–0.5 mm, and duration of 30 min. In these studies, the authors proved that this intervention was able to persistently modify the activation interplay between the vibrated muscle and its antagonists and it was correlated with an increase in motor coordination in the joints and, therefore, with a likely articular performance increase [20,48,49,50].

Another relevant piece of data in the present study is the muscular activation of different muscles of the lower extremity after receiving the combined protocol. Similar to another study [11], where they found less muscle activation in the rectus femoris, vastus lateralis, and vastus medialis after receiving focal vibration, in the present study, although the changes are not statistically significant, minor activation has also been found in the rectus femoris, biceps femoris, gluteus maximus, and erector spinae, during the squat. Another study [49] emphasized that even though muscle function had improved, no changes were observed in the amplitude of the M wave after the protocol. This suggests that improvements in muscle function are not due to a local warm-up effect, supporting the notion that the effects of vibration on muscle function are derived from enhanced corticomotor excitability. In the general sense, intracortical inhibition can be considered as a mechanism that improves muscle selection and reduces superfluous co-contractions, thereby improving the efficiency of the motor task [51]. Consequently, in the present study, peak power and peak velocity enhancement might be justified by a different motor unit recruitment and agonist–antagonist interplay as a consequence of modified motor control management [52].

The results suggest that the improvements in muscle function are not merely due to a local effect but maybe linked to central nervous system responses, supporting the use of focal vibration as a complementary tool in training programs for amateur athletes. Moreover, the slight reduction in muscle activation observed in some lower-limb muscles may indicate that this approach also helps mitigate fatigue by promoting more efficient neuromuscular function. At a clinical level, combining high-intensity focal vibration during voluntary contraction could help different athletes to optimize their results, improve the efficiency of their training, and reduce the risk of injury in their competitions. One of the limitations of this study is that this study was conducted in amateur athletes, which can limit the generalizability of the results to trained or professional athletes; another limitation is not being able to evaluate midterm focal vibration effects. Future research should also consider longitudinal studies to investigate the long-term effects of focal vibration combined with strength warm-ups in different populations and sport disciplines.

## 5. Conclusions

This study shows that the application of focal vibration at 100–180 Hz combined with voluntary contraction significantly enhances peak and mean power and velocity during squat exercises in amateur athletes in comparison with contraction in isolation, suggesting that the combination of high-frequency vibration with voluntary contraction can optimize neuromuscular performance linked to enhanced corticomotor excitability and refined motor control. In addition to optimizing neuromuscular function, this approach may serve as a valuable tool for reducing injury risk and maximizing training outcomes, particularly in less-experienced athletes who may benefit more from such interventions. Future research should explore the long-term effects of this protocol, optimize its parameters, and test its applicability across different athletic populations.

## Figures and Tables

**Table 1:** Demographic characteristics of the sample.

	Experimental Group (n = 36)	Sham Group (n = 36)	*p*
Age (years)	25 ± 5	21 ± 4	0.594
Sex	25 (69.5%) Men 11 (30.5%) Women	22 (61.1%) Men 14 (38.9%) Women	0.458
Dominance	24 (66.7%) Right 12 (33.3%) Left	28 (88.8%) Right 8 (22.2%) Left	0.293
Height (cm)	174 ± 9	173 ± 10	0.691
Weight (kg)	72.7 ± 13.8	70.3 ± 13.5	0.466
Hours of sport per week	6.3 ± 2.6	6.7 ± 3.6	0.683

Abbreviations: (cm) centimeters; (kg) kilograms.

**Table 2:** Within-group comparison of peak and mean power and peak and mean velocity.

	Experimental Group			Sham Group		
Outcome	Pre-Treatment	Post-Treatment		Pre-Treatment	Post-Treatment	
	Mean ± SD	Mean ± SD	*p*/ES/%	Mean ± SD	Mean ± SD	*p*/ES/%
Peak power (Watts)	519 ± 207	534 ± 207	*p* = 0.052 ES = 0.08 2.9%	457 ± 188	443 ± 176	*p* = 0.055 ES = 0.08 −3.2%
Mean power (Watts)	488 ± 193	512 ± 196	*p* < 0.001 ES = 0.13 4.9%	422 ± 161	421 ± 159	*p* = 0.570 ES = 0.01 −0.4%
Peak velocity (m/s)	0.91 ± 0.06	0.94 ± 0.08	*p* < 0.010 ES = 0.42 3.3%	0.92 ± 0.11	0.90 ± 0.10	*p* < 0.016 ES = 0.26 −2.2%
Mean velocity (m/s)	0.86 ± 0.06	0.90 ± 0.07	*p* < 0.001 ES = 0.66 5.9%	0.85 ± 0.07	0.84 ± 0.08	*p* = 0.367 ES = 0.07 −1.2%

Abbreviations: (m/s) meters per second; *p*: *p*-value; and ES: Cohen’s d.

**Table 3:** Between-group comparison of peak and mean power and peak and mean velocity.

Outcome	Difference Experimental Group Pre- and Post-Treatment	Difference Sham Group Pre- and Post-Treatment	
	Mean ± SD	Mean ± SD	*p*
Peak power (Watts)	14 ± 29	−14 ± 56	*p* < 0.049 ES = 0.65
Mean power (Watts)	23 ± 20	−1 ± 15	*p* < 0.034 ES:1.42
Peak velocity (m/s)	0.03 ± 0.04	−0.03 ± 0.08	*p* < 0.024 ES:0.95
Mean velocity (m/s)	0.05 ± 0.03	−0.00 ± 0.03	*p* < 0.002 ES:1.67

Abbreviations: (m/s) meters per second; *p*: *p*-value; and ES, effect size: Cohen’s d.

**Table 4:** Muscle activity (%) values in vastus medialis, vastus lateralis, rectus femoris, biceps femoris, gluteus maximus, and erector spinae longissimus muscles during the squat post-treatment and difference between pre-treatment.

Outcome	Group	RMS Pre-Treatment	RMS Post-Treatment	Difference Between Pre- and Post-Treatment			Group × Temps Interaction	
		Mean ± SD	Mean ± SD	Mean	95% CI	*p*	F	*p*
Rectus Femoris	Experimental Group	100 ± 0	94.78 ± 22.41	−5.22	−12.28; 1.83	0.144	0.004	0.952
	Sham Group	100 ± 0	95.07 ± 18.17	−4.93	−11.68; 1.83	0.150		
Vastus Lateralis	Experimental Group	100 ± 0	100.41 ± 22.86	0.41	−9.70; 10.51	0.936	0.203	0.654
	Sham Group	100 ± 0	103.57 ± 33.80	3.56	−6.11; 13.24	0.465		
Vastus Medialis	Experimental Group	100 ± 0	105.96 ± 34.19	5.96	−8.21; 20.12	0.404	0.001	0.976
	Sham Group	100 ± 0	106.26 ± 42.75	6.26	−7.67; 20.20	0.372		
Biceps Femoris	Experimental Group	100 ± 0	95.64 ± 19.48	−4.36	−11.86; 3.15	0.250	0.092	0.763
	Sham Group	100 ± 0	94.07 ± 22.13	−5.93	−13.09; 1.24	0.103		
Gluteus Maximus	Experimental Group	100 ± 0	96.07 ± 28.07	−3.93	−20.13; 12.27	0.630	2.135	0.149
	Sham Group	100 ± 0	112.37 ± 57.24	12.37	−12.27; 20.13	0.111		
Erector Spinae Longissimus	Experimental Group	100 ± 0	99.27 ± 25.48	−0.73	−13.42; 11.96	0.909	0.299	0.587
	Sham Group	100 ± 0	104.04 ± 42.21	4.04	−7.90; 15.98	0.502		

Abbreviations: RMS, root mean square; SD, standard deviation; CI, confidence interval; and *p*, *p*-value.

## Data Availability

Data from this study may be shared upon justified and reasonable request.
